# Establishing the Research Priorities of ADHD Professionals: An International Delphi Study

**DOI:** 10.1177/10870547241307739

**Published:** 2024-12-25

**Authors:** Kate Stephens, Emma Sciberras, Matthew Bisset, Ainsley Summerton, David Coghill, Christel M Middeldorp, Leanne Payne, Mark A Bellgrove, Stephen V. Faraone, Tobias Banaschewski, Jeffery H. Newcorn, Stacey D. Espinet, Iris Manor, Mohammed M.J. Alqahtani, Jeremy Varnham, Timothy J Silk

**Affiliations:** 1 Deakin University, Melbourne, VIC, Australia; 2 Murdoch Children’s Research Institute, Melbourne, VIC, Australia; 3 The University of Melbourne, Parkville, VIC, Australia; 4 The Royal Children’s Hospital, Parkville, VIC, Australia; 5 Amsterdam University Medical Center, The Netherlands; 6 Arkin Mental Health Care, Amsterdam, The Netherlands; 7 Academic Center for Child and Adolescent Psychiatry, Amsterdam, The Netherlands; 8 The University of Queensland, Brisbane, Australia; 9 Children’s Health Queensland Hospital and Health Service, Brisbane, Australia; 10 Monash University, Melbourne, VIC, Australia; 11 SUNY Upstate Medical University, Syracuse, USA; 12 University of Heidelberg, MannheimPartner Site Mannheim-Heidelberg-Ulm, Germany; 13 Icahn School of Medicine at Mount Sinai, New York, NY, USA; 14 Canadian ADHD Resource Alliance, Toronto, ON, Canada; 15 Tel-Aviv University, Israel; 16 Saudi ADHD Society, Riyadh, Saudi Arabia; 17 King Khalid University, Abha, Saudi Arabia

**Keywords:** ADHD, research, priorities

## Abstract

**Objectives:**

To determine ADHD research priorities from the perspective of ADHD professionals internationally.

**Method:**

A two-stage modified Delphi design was used. In Stage 1 (qualitative), participants listed research questions relating to ADHD that they perceived to be most important (*N* = 132). In Stage 2 (quantitative), participants were then asked to rate each research question that was deemed appropriate (able to be researched and not already addressed by research) in terms of perceived importance (*N* = 180).

**Results:**

Stage 1 generated 382 research questions with 10 broad areas identified for example, co-occurring conditions and treatment, etc. The top 20 most important questions related to ADHD in women/girls, long-term medication use, non-pharmacological interventions, ADHD measurement/rating scales, and efficacy of emotional regulation interventions.

**Conclusion:**

These results can inform an ADHD research agenda which represents the views of the individuals from major ADHD professional groups internationally. Parallel work is needed focusing on research priorities from the perspective of ADHD consumers.

Despite substantial advances in knowledge related to ADHD, there are many questions that need answers to guide clinical practice and optimize outcomes for individuals with ADHD. However, very little is known about which research questions are deemed to be most important by ADHD professionals. We aimed to understand ADHD research priorities of professionals across the world. These data are critical in shaping the strategic priorities and advocating for research funding.

To date, two small scale studies have examined ADHD research priorities (refer to Table 1). Gaynes et al. (2014) used a systematic review as a foundation to work with a group of 12 stakeholders to identify and prioritize research needs. They identified eight priorities with the majority focusing on the effectiveness of pharmacologic and psychosocial treatments for children under the age of six. Only two were focused on people aged over six, while another focused on accurate and brief standardized diagnosis and assessment.

**Table 1.** Research Priorities Identified by Gaynes et al. (2014) and Jacobson et al. (2016).

Study	Research priorities
Gaynes et al. (2014)	• Accurate, brief standardized diagnosis and assessment. • Comparative effectiveness and safety of pharmacologic treatments for children under 6 years of age. • Comparative effectiveness of different combinations of psychosocial and pharmacologic treatments for children under 6 years of age. • Comparative effectiveness of psychosocial treatment alone versus pharmacologic and combination treatments for children under 6 years of age. • Comparative long-term treatment effectiveness for people 6 years of age and older. • Relative efficacy of specific psychosocial program components for children under 6 years of age. • Identification of person-level effect modifiers for people 6 years of age and older. • Case identification and measurement of prevalence and outcomes.
Jacobson et al. (2016)	• Is there a risk that medication with methylphenidate during childhood will lead to the development of drug dependence later in life? • What are the effects of teacher support? • What are the effects of multimodal therapy? • Which of the two pharmaceuticals, atomoxetine, or methylphenidate, is most effective, with fewer side effects? • What are the effects of methylphenidate medication in substance abusers? • What are the effects of parental support programs? • What are the effects of supported conversation? • What are the effects of computer-aided working memory training? • What are the effects of psychoeducative treatment? • What are the effects of treatment of sleep disorders with melatonin?

Jacobson et al. (2016) developed a top 10 research list through a working group comprising consumers and professionals involved in the treatment of ADHD in schools, health, and corrective services. While they also identified several priorities relating to medication use, there were also priorities focused on the effects of teacher support, memory training, and treating sleep disorders. Although both studies provided some insight into where research should be directed, they were relatively small scale, and did not use a systematic approach such as the Delphi method in determining research priorities.

The Delphi Method is a structured communication technique used to gather expert opinions and achieve a consensus on complex issues through iterative rounds of questioning and feedback (Byrne et al., 2008; Crisp et al., 1997; Rudolph et al., 2009). The Delphi method typically includes 30 or more panel members, involves seeking their opinion on one or more questions and then has a summarization component in which their opinions are used in developing a survey for further comment. A study by Rudolph et al. (2009), in large group of Australian pediatricians, used a systematic two stage Delphi approach to determine the most research priorities for their clinical practice. In the first stage, the pediatricians were asked an open-ended question about their research priorities. Following qualitative coding and synthesis, the top 20 research questions were extracted and sent back to participants to rate on a 5-point Likert scale assessing importance (Rudolph et al., 2009). This design enabled a relatively large group of pediatricians (*N* = 348) to have their say about research priorities rather than relying on a small panel of representatives.

The current work sought to examine ADHD research priorities from the perspectives of individual in multiple professional groups working in ADHD. It represents the largest and most systematic effort to date to determine the research priorities of ADHD professionals by surveying members of major professional groups working in ADHD internationally.

## Methods

### Design

This cross-sectional study used a two-stage Delphi design to combine qualitative (main research questions) and quantitative questions (demographics and ratings of priorities), presented to participants in two surveys and completed electronically at two different timepoints. This method was adapted from a previous study used to establish the research priorities of Australian and American pediatricians (Rudolph et al., 2009).

### Target Population

Professionals from nine international ADHD organizations participated (90% response rate*):

- Australian ADHD Professionals Association (AADPA)
- European Network for Hyperkinetic Disorders (EUNETHYDIS)
- World Federation of ADHD
- CADDRA—Canadian ADHD Resource Alliance
- American Professional Society of ADHD and Related Disorders (APSARD)
- Asian Federation of ADHD
- Israeli Society of ADHD
- Saudi ADHD Society
- World Psychiatric Association

*A tenth organization was invited but did not participate.

### Measures

#### Stage 1 Survey

The Stage 1 survey consisted of ten questions including the main qualitative research question: “Thinking about your professional practice (e.g., clinical practice, research, and coaching), briefly list the three most important research questions relating to ADHD that (to your knowledge) have not been adequately addressed?”. Additionally, quantitative questions were included regarding sex, age, developmental age of primary focus (e.g., childhood and adolescence), working hours (e.g., full time and part time), if currently involved in research, practice type (e.g., private and public), region of practice (e.g., urban, suburban, and rural), location of main practice, member of which ADHD organization.

#### Stage 2 survey

The Stage 2 survey listed the unique research questions identified in Stage 1 in random order. Participants were asked to rate each research priority in relation to how important it is for their professional practice on a 5-point Likert scale (e.g., “please rate whether each research priority is *Not Important*, *Of Little Importance*, *Moderately Important*, *Important*, or *Very Important/Essential* for your professional practice”). An open-ended question was added to identify any other research priorities that were not already included (“Are there other questions you believe are important research questions that haven’t been covered above? If yes, thinking about your professional practice (e.g., clinical practice, research, and coaching), briefly list up to three of the most important research questions relating to ADHD that (to your knowledge) have not been adequately addressed”). The same demographic questions from Stage 1 were also collected at Stage 2 (e.g., sex, age, profession, developmental age of primary focus, working hours, if currently involved in research, practice type, region of practice, location of main practice, and member of which ADHD organization).

### Method

The project was approved by the Deakin University Human Research Ethics Committee (2019-340). Recruitment was conducted in three stages. First, an invitation was sent to contacts at each ADHD organization seeking their agreement to distribute information about the study via their organization’s email distribution list. If the contact agreed, they were asked to complete and return a letter of support to the investigator team.

#### Stage 1

A Stage 1 invitation email with a description and link to the Plain Language Statement and Stage 1 survey was then sent to potential participants via each participating organization’s email distribution list, for participants to opt into the study. The Stage 1 survey remained available for completion for a period of 6 months with general reminder emails being sent via each organization’s email distribution list 2, 4, and 6 weeks after the initial Stage 1 invitation email was sent.

#### Stage 2

Responses from the Stage 1 survey were then collated by the research team and the research priorities identified by participants were synthesized into a comprehensive list of research questions. Questions were either rated as appropriate and should be included in the Stage 2 survey, already addressed by research and should be excluded (typically questions on prevalence of co-occurring conditions with ADHD, for example “What is the prevalence of autism spectrum disorder in those with ADHD?”), unable to be addressed by research and should be excluded (less tangible questions such as “What do we still need to know about the comorbidity?” and “How can more effective medications be discovered?”), or unclear. The rating was conducted by a research steering committee (*N* = 12) consisting of members from the authorship team (*n* = 5) and contacts from participating international ADHD organizations (*n* = 7), with each question required to be endorsed by at least 80% of the research steering committee for the question to be included in the Stage 2 survey.

A Stage 2 invitation email and Plain Language Statement was then sent to participating ADHD organizations for distribution to their members along with a link to complete the Stage 2 survey. Reminder emails were sent via each organization’s email distribution list 2-, 4-, and 6-week after the initial Stage 2 email was sent.

#### Stage 2a

An additional 96 suggested research questions were submitted by professionals in answer to the Stage 2 open-ended question, with 25 of these rated as appropriate using the same approach in Stage 1. These questions identified additional areas of interest such as women/girls with ADHD, understanding clinicians’ beliefs/knowledge, and the most effective approach to training. These additional questions were then sent to all participants who completed Stage 2.

### Statistical Analysis

The Stage 1 survey was completed by 128 participants and generated 382 research questions. From this, a list of 124 unique questions were identified (i.e., removing duplicates or questions where the meaning was unclear). Two raters completed qualitative coding of the generated questions to identify the main categories of research that they covered.

Stata SE 17.0 was used to complete data analysis. Data from Stage 2 and Stage 2a surveys were merged. Participants were required to have responded to at least 80% of research questions to have responses included in the analysis. Where there were duplicate data from the same survey, the most complete response was retained. If a participant had completed the same survey more than once (genuine duplicate), the first response was retained. In total, 20 duplicates were removed. There were 253 responses included in the final analysis. For each research question, the mean, standard error, and 95% confidence interval was calculated. Research questions were then ranked in order of importance from 1 to 94 based on the mean score. The frequency of each response (0 = not important, 1 = of little importance, 2 = moderately important, 3 = important, and 4 = very important) for each research question was also calculated.

## Results

### Sample characteristics

Table 2 displays the sample characteristics for each survey. Demographics were similar for Stages 1 and 2 with the main difference being there were fewer male respondents at Stage 2. Australia, America and Canada had the highest number of respondents at both stages. Over half the respondents for each stage were psychiatrists, psychologists, neuropsychologists, or pediatricians/general practitioners. Childhood was the most reported focus of practice for Stage 1 and 2, while adulthood was the most frequently reported focus of practice for Stage 2a.

**Table 2.** Sample Characteristics.

Sample characteristics	Stage 1	Stage 2	Stage 2a
	*n* (%)	*n (%)*	*n (%)*
	(*N* = 128)	(*N* = 180)	(*N* = 73)
Male	71 (55.5)	62 (35.6)	21 (28.8)
Age (years), *M* (*SD*)			
25–34	5 (3.9)	14 (7.8)	2 (2.7)
35–44	18 (14.1)	30 (17.1)	11 (15.1)
45–54	40 (31.3)	58 (33.1)	22 (30.1)
55–64	38 (29.7)	40 (22.9)	21 (28.8)
65 and over	27 (21.1)	28 (16.0)	17 (23.3)
Profession			
Psychiatrist	49 (38.6)	35 (24.1)	12 (16.4)
Psychologist/neuropsychologist	23 (18.1)	42 (24.1)	17 (23.3)
Pediatrician/general practitioner	17 (13.4)	29 (16.7)	14 (19.2)
Researcher	16 (12.6)	18 (10.3)	5 (6.9)
Coach	5 (3.9)	12 (6.9)	5 (6.9)
Nurse practitioner	3 (2.4)	4 (2.3)	1 (1.4)
Social worker	2 (1.6)	6 (3.4)	2 (2.7)
Student	2 (1.6)	0 (0.0)	0 (0.0)
Other	9 (7.1)	27 (15.5)	15 (20.6)
Focus of practice—developmental age			
Childhood (up to 12 years)	51 (40.2)	63 (37.1)	19 (26.0)
Adolescence (13–17 years)	15 (11.8)	20 (11.8)	5 (6.9)
Early adulthood (18–24 years)	15 (11.8)	22 (12.9)	8 (10.9)
Adulthood (25 years and over)	42 (33.1)	65 (38.2)	41 (56.2)
Working hours in field of ADHD			
Full time	77 (60.6)	97 (55.4)	33 (45.2)
Part time	47 (37.0)	73 (41.7)	38 (52.1)
Other	3 (2.4)	5 (2.9)	2 (2.7)
Involved in research	74 (58.3)	73 (41.7)	26 (35.6)
Type of ADHD practice			
Private practice	60 (47.2)	105 (58.7)	53 (72.6)
Academic	45 (35.4)	45 (25.1)	13 (17.8)
Public hospital	23 (18.1)	19 (10.6)	11 (15.1)
Community center	15 (11.8)	19 (10.6)	6 (8.2)
Region of practice			
Urban	74 (58.3)	71 (53)	16 (33.3)
Suburban	15 (11.8)	39 (29.1)	18 (37.5)
Rural	6 (4.7)	10 (7.5)	6 (12.5)
Country of practice			
Australia	26 (20.3)	51 (30.0)	18 (25.0)
United States of America	21 (16.4)	35 (21.2)	18 (25.0)
Canada	15 (11.7)	36 (20.6)	20 (27.8)
Germany	12 (9.4)	9 (5.3)	3 (4.2)
Israel	7 (5.5)	3 (1.8)	0 (0)
United Kingdom	5 (3.9)	4 (2.4)	1 (1.4)
Brazil	4 (3.1)	0 (0.0)	0 (0.0)
China	3 (2.3)	0 (0.0)	0 (0.0)
Norway	3 (2.3)	0 (0.0)	0 (0.0)
Saudi Arabia	3 (2.3)	5 (2.9)	2 (2.8)
Netherlands	0 (0.0)	6 (3.5)	1 (1.4)
Switzerland	0 (0.0)	3 (1.8)	1 (1.4)
Other	14 (10.9)	20 (11.8)	8 (11.1)

#### Stage 1

From the qualitative coding, 59 categories were included with many questions covering several categories at once (e.g., specific age group and comorbidities, etc.). Half of the questions addressed treatment (47.58%), and a third addressed comorbidities (32.26%) or medication (31.45%). Although some research questions specified specific age groups (e.g., children or adults), three quarters of the questions did not have a specific age focus.

#### Stage 2

In Stage 2, the final 94 questions were ranked from most important to least important based on ratings provided by professionals. Table 3 displays the most important 20 research questions. Topics included: ADHD in women/girls; long term medication use; ADHD measure/rating scales; efficacy and long-term outcomes of non-pharmacological interventions; sleep; and protective factors. A full list ranking all 94 questions along with item frequencies is included in Supplemental Table 1.

**Table 3.** Top 20 Ranked Research Questions.

Ranking	Question	Mean	*SD*	95% CI
1	How can ADHD rating scales be adapted to better measure ADHD in individuals with ADHD with high intelligence who may be using successful coping strategies to manage their symptoms?	3.43	0.10	[3.23, 3.63]
2	How can we best identify and treat ADHD in women/girls?	3.38	0.08	[3.22, 3.54]
3	What are protective factors for people with ADHD and how can they be promoted?	3.37	0.10	[3.17, 3.57]
4	How can the future DSM criteria for ADHD be expanded to better capture symptoms domains relevant for adults?	3.36	0.11	[3.14, 3.57]
5	What is the course of ADHD in females across the life cycle?	3.33	0.10	[3.13, 3.53]
6	What are the most effective interventions for emotional dysregulation in ADHD?	3.32	0.07	[3.19, 3.45]
7	Do hormones play a role in the symptom emergence and related functional impairments in women with ADHD?	3.32	0.10	[3.11, 3.52]
8	What are the long-term effects of stimulant and non-stimulant medications on behavior, cognition, and the brain?	3.28	0.06	[3.15, 3.40]
9	What are the long-term benefits and side effects of ADHD medication?	3.27	0.06	[3.15, 3.40]
10	What is the best treatment for sleep difficulties in ADHD and the prevention of health problems?	3.22	0.06	[3.09, 3.35]
11	What are the longitudinal trajectories of medicated vs. non-medicated children with ADHD?	3.20	0.07	[3.06, 3.33]
12	Which pharmacological interventions are effective for treating emotion dysregulation in individuals with ADHD?	3.17	0.07	[3.04, 3.30]
13	What are the core features of effective non-pharmacological interventions at different ages (especially in children)?	3.14	0.12	[2.90, 3.38]
14	How can more robust measures of attentional profiles and treatment response be developed to inform ADHD-related diagnosis and treatment?	3.13	0.07	[3.00, 3.27]
15	What is the most efficient parent training approach for ADHD?	3.12	0.07	[2.98, 3.25]
16	What are the long-term outcomes of psychosocial treatments for ADHD?	3.10	0.07	[2.97, 3.23]
17	What are the most effective and efficient ways to screen for ADHD?	3.08	0.07	[2.93, 3.23]
18	Can medication interventions be tailored to promote cognitive and executive functioning among individuals with brain-fog/ADHD-I/sluggish cognitive tempo-like symptoms?	3.07	0.12	[2.83, 3.31]
19	What are the most effective treatments for ADHD with comorbid mood disorder (and does this differ by age)?	3.06	0.06	[2.93, 3.18]
20	What are clinicians’ (e.g., psychiatrists, psychologists, pediatricians, and physicians) beliefs, knowledge, and confidence in treating ADHD?	3.06	0.13	[2.80, 3.31]

## Discussion

This study sought to determine the ADHD research priorities of professionals belonging to multiple international ADHD professional groups. After generating an initial list of 382 research questions in Stage 1, and ranking 94 questions in Stage 2, the top 20 most important research questions covered topics related to ADHD in women/girls, long term medication use, ADHD measurement/rating scales, efficacy of interventions for emotional regulation, sleep, and protective factors.

Three questions relating to ADHD in women/girls were in the top ten most important research questions. This differed to the previous two studies examining research priorities where this area of research was not identified at all (Gaynes et al., 2014; Jacobson et al., 2016). Historically, research into ADHD has predominantly focused on males, with females being largely understudied (for a full review see Hinshaw et al., 2022). Although males are diagnosed more frequently in childhood, this ratio becomes closer to equal in adulthood (Kessler et al., 2006; Nussbaum, 2012). In recent years there has been an increased focus on ADHD in women and girls (Hinshaw et al., 2022), therefore it is not surprising that our sample of ADHD professionals ranked understanding the life course of ADHD and identifying and treating it effectively in women and girls as an important priority. A recent systematic review by Hinshaw et al. (2022) also highlighted a key direction for research into ADHD in females is the study of biological and psychosocial changes related to life changes such as puberty, childbirth/child rearing, and perimenopause and menopause. Understanding how hormones play a role in symptom emergence and functioning difficulties in females with ADHD was also ranked in the top ten most important questions in the current study. Our findings show that ADHD professionals identify this as an important area for research that requires further study.

Medication use was another area where several questions ranked in the top 20 most important. Participants identified long term effects and benefits, as well as the side effects as important research priorities. Both Gaynes et al (2014) and Jacobson et al. (2016) previously identified the efficacy of pharmacological treatment as a research priority, however most of the pharmacological research questions within the top 20 ranked priorities for the current study were specifically interested in longer term use. While recent reviews suggest that ADHD medications are safe and effective (Chang et al., 2019; Coghill et al., 2023; Krinzinger et al., 2019; Man et al., 2023), they have also shown that further high-quality data measuring longer term outcomes and effectiveness is needed (Hennissen et al., 2017; Kovshoff et al., 2016).

Non-pharmacological interventions were also ranked as an important priority in the current study, with four questions in the top 20. Like the pharmacological research questions, there was an interest in the longer-term outcomes of psychosocial treatments. The other questions focused on the core features of effective interventions at different ages, most efficient parent training approaches and effective interventions for emotional dysregulation. This area of research priority is consistent with the previous studies conducted by Gaynes et al. (2014) and Jacobson et al. (2016), however, theirs had a stronger focus on children. One recent review of nonpharmacological randomized control trials showed significant efficacy and good tolerability of several non-pharmacological interventions including cognitive behavior therapy, mindfulness, and dialectic behavioral therapy in adults with ADHD (Nimmo-Smith et al., 2020). However, there appears to still be a need for longer-term outcomes.

While the professionals who participated in the current study have helped to identify several important research priority areas, it is also important to note the need for concurrent research focusing on the priorities of ADHD consumers. A recent systematic review conducted to understand the unmet needs of healthcare consumers with ADHD found there were very few studies published in the last 10 years that sought insight from consumers about their needs (Bisset et al., 2023). Their findings showed that ADHD consumers have many unmet needs relating to multiple areas including ADHD-related education and training for consumers, professionals and educators, clinical care, access to service/supports, and accommodations in schools. It is important to understand whether consumer research priorities are similar to ADHD professionals’ priorities, or whether there are additional areas identified that need more focus.

A strength of this study is that it is the largest to examine ADHD research priorities and the first to examine it at an international level. It is also the first to utilize a two-stage systematic approach. Previous studies used small samples in one location, making it difficult to know whether those priorities were also important to ADHD professionals generally. A limitation of this study is that most respondents to both surveys were from Australia, America, and Canada. This limits the extent these results can be generalized to other countries which may have different approaches or priorities when it comes to the identification and treatment of ADHD. Similarly, the sample was comprised predominately of people working in a clinical capacity with ADHD, with only 6%-12% [depending on Stage] identified as researchers. Whilst 35% to 58% of the respondents did report being involved in research, this composition may have influenced the identified priorities (such as greater focus on diagnosis and treatment, and less focus on biological mechanisms). Future research should aim to get a wider distribution of participants, to gain a more representative understanding of worldwide ADHD research priorities. Another limitation is that the survey response rate is unknown due to the individual organizations being responsible for distributing the survey to their members.

## Conclusions

According to the perspectives of ADHD professionals, ADHD in females, long-term pharmacological benefits and side effects, along with effective non-pharmacological interventions and outcomes were highlighted as important priorities. Future research adopting a longitudinal, life course approach, may be best to address some of the top-ranked research priorities identified in this study. The results of this study may be used to form an ADHD research agenda which represents the views of a large proportion of professionals associated with these ADHD groups. There is also a need for parallel work focusing on research priorities from the perspectives of ADHD consumers.

## Supplemental Material

sj-docx-1-jad-10.1177_10870547241307739 – Supplemental material for Establishing the Research Priorities of ADHD Professionals: An International Delphi Study Supplemental material, sj-docx-1-jad-10.1177_10870547241307739 for Establishing the Research Priorities of ADHD Professionals: An International Delphi Study by Kate Stephens, Emma Sciberras, Matthew Bisset, Ainsley Summerton, David Coghill, Christel M Middeldorp, Leanne Payne, Mark A Bellgrove, Stephen V. Faraone, Tobias Banaschewski, Jeffery H. Newcorn, Stacey D. Espinet, Iris Manor, Mohammed M.J. Alqahtani, Jeremy Varnham and Timothy J Silk in Journal of Attention Disorders
