# Supplementary material for: Establishing the Research Priorities of ADHD Professionals: An International Delphi Study
Source: J Atten Disord. 2024 Dec 25;29(5):303–11. doi: 10.1177/10870547241307739 (PMC11800688; doi:10.1177/10870547241307739)
Supplement: sj-docx-1-jad-10.1177_10870547241307739 – Supplemental material for Establishing the Research Priorities of ADHD Professionals: An International Delphi Study [file sj-docx-1-jad-10.1177_10870547241307739.docx]

**Supplementary Table 2.**

*Full list of final research priorities ranked by importance (with frequencies)*

*(Where 0 = Not important, 1 = Of little importance, 2 = Moderately important, 3 = Important, 4 = Very important/essential)*

| **Question** | **0** | **1** | **2** | **3** | **4** | **M** | **SD** | **95% lower** | **95% upper** |
| --- | --- | --- | --- | --- | --- | --- | --- | --- | --- |
| How can ADHD rating scales be adapted to better measure ADHD in individuals with ADHD with high intelligence who may be using successful coping strategies to manage their symptoms? | 0 | 3 | 8 | 16 | 45 | 3.43 | 0.10 | 3.23 | 3.63 |
| How can we best identify and treat ADHD in women/girls? | 0 | 0 | 8 | 29 | 36 | 3.38 | 0.08 | 3.22 | 3.54 |
| What are protective factors for people with ADHD and how can they be promoted? | 1 | 2 | 5 | 26 | 39 | 3.37 | 0.10 | 3.17 | 3.57 |
| How can the future DSM criteria for ADHD be expanded to better capture symptoms domains relevant for adults? | 0 | 4 | 11 | 13 | 45 | 3.36 | 0.11 | 3.14 | 3.57 |
| What is the course of ADHD in females across the life cycle? | 1 | 0 | 12 | 20 | 39 | 3.33 | 0.10 | 3.13 | 3.53 |
| What are the most effective interventions for emotional dysregulation in ADHD? | 2 | 8 | 14 | 60 | 93 | 3.32 | 0.07 | 3.19 | 3.45 |
| Do hormones play a role in the symptom emergence and related functional impairments in women with ADHD? | 0 | 3 | 17 | 17 | 41 | 3.32 | 0.10 | 3.11 | 3.52 |
| What are the long-term effects of stimulant and non-stimulant medications on behaviour, cognition and the brain? | 1 | 6 | 20 | 67 | 84 | 3.28 | 0.06 | 3.15 | 3.40 |
| What are the long-term benefits and side effects of ADHD medication? | 0 | 7 | 28 | 53 | 91 | 3.27 | 0.06 | 3.15 | 3.40 |
| What is the best treatment for sleep difficulties in ADHD and the prevention of health problems? | 0 | 7 | 30 | 59 | 83 | 3.22 | 0.06 | 3.09 | 3.35 |
| What are the longitudinal trajectories of medicated vs. non-medicated children with ADHD? | 2 | 8 | 24 | 64 | 81 | 3.20 | 0.07 | 3.06 | 3.33 |
| Which pharmacological interventions are effective for treating emotion dysregulation in individuals with ADHD? | 0 | 9 | 29 | 65 | 77 | 3.17 | 0.07 | 3.04 | 3.30 |
| What are the core features of effective non-pharmacological interventions at different ages (especially in children)? | 2 | 3 | 11 | 23 | 33 | 3.14 | 0.12 | 2.90 | 3.38 |
| How can more robust measures of attentional profiles and treatment response be developed to inform ADHD-related diagnosis and treatment? | 0 | 10 | 35 | 55 | 79 | 3.13 | 0.07 | 3.00 | 3.27 |
| What is the most efficient parent training approach for ADHD? | 2 | 8 | 33 | 61 | 76 | 3.12 | 0.07 | 2.98 | 3.25 |
| What are the long-term outcomes of psychosocial treatments for ADHD? | 1 | 8 | 32 | 69 | 69 | 3.10 | 0.07 | 2.97 | 3.23 |
| What are the most effective and efficient ways to screen for ADHD? | 3 | 10 | 35 | 53 | 78 | 3.08 | 0.07 | 2.93 | 3.23 |
| Can medication interventions be tailored to promote cognitive and executive functioning among individuals with brain-fog/ADHD-I/sluggish cognitive tempo-like symptoms? | 2 | 4 | 11 | 25 | 30 | 3.07 | 0.12 | 2.83 | 3.31 |
| What are the most effective treatments for ADHD with comorbid mood disorder (and does this differ by age)? | 0 | 9 | 33 | 77 | 61 | 3.06 | 0.06 | 2.93 | 3.18 |
| What are clinicians' (e.g., psychiatrists, psychologists, paediatricians, physicians) beliefs, knowledge, and confidence in treating ADHD? | 1 | 7 | 12 | 19 | 33 | 3.06 | 0.13 | 2.80 | 3.31 |
| What alternatives to medication are effective for treating ADHD? | 2 | 12 | 36 | 54 | 75 | 3.05 | 0.07 | 2.90 | 3.20 |
| What are the most effective methods/ strategies for teaching primary school aged children with ADHD in the classroom? | 4 | 9 | 38 | 54 | 73 | 3.03 | 0.08 | 2.88 | 3.18 |
| What approaches can be used to best support children and adolescents with ADHD in the school setting? | 2 | 11 | 32 | 70 | 65 | 3.03 | 0.07 | 2.89 | 3.17 |
| How can intervention programs promote coping strategies in parents of young people with ADHD in order to improve family interactions? | 2 | 15 | 39 | 42 | 78 | 3.02 | 0.08 | 2.86 | 3.17 |
| What are the most effective interventions for substance use and addiction in patients with ADHD? | 0 | 15 | 31 | 68 | 64 | 3.02 | 0.07 | 2.88 | 3.16 |
| Can interventions for young people with ADHD protect against later offending behaviours? | 2 | 11 | 36 | 66 | 64 | 3.00 | 0.07 | 2.86 | 3.14 |
| Can interventions for young people with ADHD protect against later substance abuse? | 2 | 10 | 40 | 63 | 65 | 2.99 | 0.07 | 2.85 | 3.13 |
| What is the nature of the association between ADHD, sleep difficulties and the circadian system? | 1 | 13 | 38 | 64 | 64 | 2.98 | 0.07 | 2.84 | 3.12 |
| What is the prevalence of undiagnosed ADHD in first presentation of depression or anxiety in adulthood? | 2 | 11 | 42 | 56 | 67 | 2.98 | 0.07 | 2.84 | 3.13 |
| How can services be developed to address the unmet needs of individuals with ADHD? | 3 | 12 | 38 | 57 | 68 | 2.98 | 0.08 | 2.83 | 3.13 |
| What is the economic burden of untreated ADHD? | 1 | 7 | 15 | 20 | 30 | 2.97 | 0.12 | 2.72 | 3.22 |
| Are there ways to predict an individual's response to ADHD medication? | 3 | 11 | 43 | 55 | 66 | 2.96 | 0.08 | 2.81 | 3.10 |
| Do stimulant medications impact on pregnancy and breastfeeding? | 1 | 6 | 19 | 16 | 30 | 2.94 | 0.13 | 2.69 | 3.20 |
| What gaps in ADHD-related knowledge exist for clinicians and teachers? | 6 | 15 | 35 | 50 | 72 | 2.94 | 0.08 | 2.77 | 3.10 |
| What are the most effective strategies to support patients to maximise the benefits of their ADHD medication? | 1 | 13 | 43 | 61 | 60 | 2.93 | 0.07 | 2.79 | 3.07 |
| What longitudinal adjustments to daily stimulant medication doses are needed to maintain treatment efficacy for up to 10 years? | 3 | 14 | 42 | 61 | 59 | 2.89 | 0.08 | 2.74 | 3.04 |
| What is the efficacy of combining stimulant and non-stimulant medications for treating ADHD? | 3 | 14 | 42 | 59 | 59 | 2.89 | 0.08 | 2.74 | 3.04 |
| How does ADHD affect the self-concept of adolescents and adults? | 2 | 13 | 45 | 61 | 56 | 2.88 | 0.07 | 2.74 | 3.03 |
| How can technology be used to optimise outcomes for individuals with ADHD? | 2 | 15 | 48 | 54 | 60 | 2.87 | 0.08 | 2.72 | 3.02 |
| What are the predictors of response to different ADHD-related interventions? | 2 | 13 | 45 | 70 | 49 | 2.84 | 0.07 | 2.70 | 2.98 |
| What psychosocial interventions are efficacious for preschool-aged children? | 8 | 13 | 42 | 61 | 54 | 2.79 | 0.08 | 2.62 | 2.95 |
| What is the emotional impact related to poor educational outcomes in people with ADHD? | 1 | 7 | 19 | 26 | 20 | 2.78 | 0.12 | 2.55 | 3.01 |
| What neurological changes (assessed by fMRI, EEG and neuropsychological testing) occur with optimal doses of ADHD medication? | 10 | 10 | 47 | 56 | 57 | 2.78 | 0.08 | 2.61 | 2.94 |
| Do cognitive deficits associated with ADHD make a significant independent contribution to overall impairment, and how can these be treated? | 4 | 15 | 48 | 64 | 49 | 2.77 | 0.08 | 2.62 | 2.92 |
| What factors influence primary care physicians' decisions to prescribe or withhold stimulant medications for adults with ADHD? | 2 | 22 | 43 | 60 | 52 | 2.77 | 0.08 | 2.62 | 2.92 |
| Can biology-related diagnostic procedures be developed for ADHD? | 5 | 19 | 46 | 50 | 57 | 2.76 | 0.08 | 2.60 | 2.93 |
| Can excessive doses of stimulant medication cause neuronal death and does this worsen ADHD symptoms? | 8 | 13 | 46 | 63 | 49 | 2.74 | 0.08 | 2.58 | 2.90 |
| What are the long-term effects of brain training (i.e. attentional/memory/ cognitive training) for individuals with ADHD? | 3 | 20 | 47 | 61 | 48 | 2.73 | 0.08 | 2.58 | 2.88 |
| What is the efficacy and safety of ADHD medication in pre-schoolers? | 5 | 24 | 42 | 53 | 55 | 2.72 | 0.08 | 2.56 | 2.89 |
| What are the neurobiological substrates of altered motivation in ADHD? | 4 | 20 | 50 | 54 | 51 | 2.72 | 0.08 | 2.56 | 2.87 |
| What type of ADHD-related education obtains the best results for reducing stigma and improving inclusion? | 2 | 24 | 45 | 60 | 48 | 2.72 | 0.08 | 2.56 | 2.87 |
| Should antidepressant or stimulant medications be first-line treatment for individuals with ADHD and comorbid depressive symptoms (and does this differ by age)? | 5 | 20 | 42 | 66 | 45 | 2.71 | 0.08 | 2.55 | 2.86 |
| What is the efficacy of psychosocial interventions for older adults with ADHD? | 3 | 20 | 44 | 69 | 41 | 2.71 | 0.08 | 2.56 | 2.85 |
| Do ADHD interventions (pharmacological and/or non-pharmacological) improve autism symptoms over time for children with comorbid ADHD and autism? | 2 | 23 | 48 | 59 | 47 | 2.70 | 0.08 | 2.55 | 2.86 |
| What is the best way to provide education on available and efficacious treatment options for ADHD? | 5 | 22 | 47 | 51 | 53 | 2.70 | 0.08 | 2.54 | 2.87 |
| Can alternative activity-based learning for young people with ADHD be provided in the current education system? | 8 | 18 | 46 | 53 | 52 | 2.69 | 0.09 | 2.53 | 2.86 |
| What biomarkers exist for ADHD? | 5 | 29 | 41 | 46 | 57 | 2.68 | 0.09 | 2.51 | 2.85 |
| What are the effects of stigma on individuals with ADHD and their families? | 2 | 18 | 58 | 65 | 37 | 2.65 | 0.07 | 2.51 | 2.79 |
| How can medication adherence be improved for individuals with ADHD? | 5 | 20 | 50 | 62 | 42 | 2.65 | 0.08 | 2.49 | 2.80 |
| Does the use of measurement-based care help to optimise outcomes for individuals with ADHD? | 4 | 28 | 45 | 53 | 49 | 2.64 | 0.08 | 2.48 | 2.81 |
| What factors affect physicians' treatment choices for children and young people with ADHD? | 5 | 28 | 44 | 58 | 41 | 2.58 | 0.08 | 2.42 | 2.74 |
| Does treatment of ADHD improve risk of vascular dementias, or does it hasten and exacerbate the developmental course? | 3 | 9 | 23 | 19 | 19 | 2.58 | 0.13 | 2.31 | 2.84 |
| What is the efficacy of 'third wave' cognitive behavioural therapies such as Acceptance and Commitment Therapy in individuals with ADHD? | 3 | 6 | 24 | 26 | 14 | 2.58 | 0.12 | 2.34 | 2.81 |
| Can biomarkers differentiate ADHD from other disorders across the life cycle? | 7 | 25 | 51 | 49 | 45 | 2.56 | 0.09 | 2.40 | 2.73 |
| How can assessment in the clinic and measures from the neuropsychology lab be improved so as to increase the degree of association between the two? | 1 | 13 | 21 | 20 | 17 | 2.54 | 0.13 | 2.29 | 2.80 |
| Which interventions are the most efficacious, tolerated and safe for individuals with ADHD and psychosis? | 6 | 27 | 54 | 48 | 43 | 2.53 | 0.08 | 2.37 | 2.70 |
| What is the efficacy and safety of ADHD medication in the elderly? | 8 | 32 | 44 | 50 | 45 | 2.51 | 0.09 | 2.34 | 2.69 |
| How does ADHD affect the elderly and can it be distinguished from dementia? | 9 | 25 | 48 | 59 | 37 | 2.51 | 0.08 | 2.34 | 2.67 |
| Is low dopamine a predictor of risk-taking behaviour in individuals with ADHD? | 3 | 11 | 19 | 27 | 13 | 2.49 | 0.13 | 2.24 | 2.75 |
| What is the efficacy, tolerability and safety of combining stimulant medications with antipsychotic medications when treating ADHD? | 8 | 25 | 55 | 53 | 38 | 2.49 | 0.08 | 2.33 | 2.66 |
| What is the prevalence of ADHD Inattentive Presentations across genders? | 1 | 10 | 27 | 24 | 11 | 2.47 | 0.11 | 2.24 | 2.69 |
| What is the best way to distinguish between ADHD and personality disorders? | 5 | 31 | 53 | 58 | 33 | 2.46 | 0.08 | 2.30 | 2.62 |
| What is the best management program to support children with ADHD and language disorder? | 7 | 24 | 58 | 59 | 30 | 2.46 | 0.08 | 2.30 | 2.61 |
| What is the association between stimulant medication and damage to dopaminergic neurons due to excitotoxicity in ADHD? | 5 | 35 | 51 | 50 | 38 | 2.45 | 0.08 | 2.29 | 2.62 |
| What is the efficacy of GABA supplements in the initial treatment of impulsivity and emotional dysregulation in children with ADHD? | 9 | 26 | 50 | 67 | 28 | 2.44 | 0.08 | 2.28 | 2.60 |
| Is there a gender disparity in age of diagnosis of ADHD? | 6 | 9 | 20 | 24 | 13 | 2.40 | 0.14 | 2.13 | 2.68 |
| What is the prevalence of chronic somatic diseases in those with ADHD? | 5 | 34 | 57 | 51 | 32 | 2.40 | 0.08 | 2.24 | 2.55 |
| What is the accuracy of the media's portrayal of ADHD and its treatment? | 5 | 28 | 67 | 50 | 29 | 2.39 | 0.08 | 2.24 | 2.54 |
| What is the prevalence of binge eating in those with ADHD? | 10 | 33 | 52 | 49 | 35 | 2.37 | 0.09 | 2.20 | 2.54 |
| What is the later prevalence of adult ADHD for those with identified learning disability at school-age? | 4 | 30 | 72 | 46 | 26 | 2.34 | 0.07 | 2.19 | 2.48 |
| What is the impact of language disorder in children with ADHD? | 10 | 27 | 64 | 55 | 23 | 2.30 | 0.08 | 2.15 | 2.46 |
| What are practitioners' beliefs about adult-onset ADHD? | 11 | 36 | 57 | 40 | 36 | 2.30 | 0.09 | 2.13 | 2.47 |
| Is there an increase in gender dysphoria among individuals with ADHD when compared to individuals without ADHD? | 5 | 15 | 19 | 24 | 9 | 2.24 | 0.13 | 1.97 | 2.50 |
| Can an electroencephalogram signature be used to validate an ADHD diagnosis? | 16 | 36 | 58 | 41 | 28 | 2.16 | 0.09 | 1.99 | 2.34 |
| Can we develop clinically relevant predictive algorithms for persistence / remission of ADHD? | 14 | 45 | 47 | 46 | 28 | 2.16 | 0.09 | 1.99 | 2.34 |
| How to differentiate ADHD from neurological conditions (e.g., Restless Legs Syndrome) in adults aged 50+? | 13 | 41 | 56 | 47 | 22 | 2.13 | 0.08 | 1.97 | 2.30 |
| What are the neuro-immune biological diatheses that contribute to ADHD? | 10 | 11 | 22 | 19 | 10 | 2.11 | 0.15 | 1.82 | 2.40 |
| What is the efficacy of neurofeedback with the different EEG-defined subtypes of ADHD? | 17 | 43 | 52 | 41 | 27 | 2.10 | 0.09 | 1.92 | 2.28 |
| Should medication strategies in the presence of BFRB's (body-focused repetitive behaviours) be different from typical strategies? | 7 | 13 | 27 | 18 | 8 | 2.10 | 0.13 | 1.83 | 2.36 |
| Can the Research Domain Criteria (Rdoc) framework help with research and practice of ADHD? | 6 | 19 | 23 | 13 | 11 | 2.06 | 0.14 | 1.78 | 2.33 |
| What is the value of Coherence vs regular neurofeedback in people with ADHD? | 13 | 28 | 17 | 10 | 4 | 1.50 | 0.13 | 1.24 | 1.76 |
| What is the efficacy and safety of traditional Chinese medicine in treating ADHD? | 32 | 69 | 49 | 16 | 12 | 1.48 | 0.08 | 1.32 | 1.64 |
| How useful are patient derived hiPSC based neuronal cell models in ADHD? | 22 | 21 | 21 | 7 | 1 | 1.22 | 0.12 | 0.98 | 1.47 |
| Does the screening and treatment for persistent primitive reflexes lead to better clinical outcomes for individuals with ADHD? | 25 | 31 | 9 | 3 | 3 | 0.99 | 0.12 | 0.74 | 1.23 |
